# Streamlined Genetic Manipulation of Diverse *Bacteroides* and *Parabacteroides* Isolates from the Human Gut Microbiota

**DOI:** 10.1128/mBio.01762-19

**Published:** 2019-08-13

**Authors:** Leonor García-Bayona, Laurie E. Comstock

**Affiliations:** a Division of Infectious Diseases, Brigham and Women’s Hospital, Harvard Medical School, Boston, Massachusetts, USA; The Forsyth Institute; Institut Pasteur; University of Chicago

**Keywords:** *Bacteroides*, microbiota, mutant construction, vectors

## Abstract

Studies of the gut microbiota have dramatically increased in recent years as the importance of this microbial ecosystem to human health and disease is better appreciated. The *Bacteroidales* are the most abundant order of bacteria in the healthy human gut and induce both health-promoting and disease-promoting effects. There are more than 55 species of gut *Bacteroidales* with extensive intraspecies genetic diversity, especially in regions involved in the synthesis of molecules that interact with other bacteria, the host, and the diet. This property necessitates the study of diverse species and strains. In recent years, the genetic toolkit to study these bacteria has greatly expanded, but we still lack a facile system for creating deletion mutants and allelic replacements in diverse strains, especially with the rapid increase in resistance to the two antibiotics used for genetic manipulation. Here, we present a new versatile and highly efficient vector suite that allows the creation of allelic deletions and replacements in multiresistant strains of *Bacteroides* and *Parabacteroides* using a gain-of-function system based on polysaccharide utilization. These vectors also allow for easy counterselection independent of creating a mutant background strain, using a toxin from a type VI secretion system of Bacteroides fragilis. Toxin production during counterselection is induced with one of two different molecules, providing flexibility based on strain phenotypes. This family of vectors greatly facilitates functional genetic analyses and extends the range of gut *Bacteroidales* strains that can be genetically modified to include multiresistant strains that are currently genetically intractable with existing genetic tools.

## Abstract (precis)

We have entered an era when studies of the gut microbiota are transitioning from basic questions of composition and host effects to understanding the microbial molecules that underlie compositional shifts and mediate health and disease processes. The importance of the gut *Bacteroidales* to human health and disease and their potential as a source of engineered live biotherapeutics make these bacteria of particular interest for in-depth mechanistic study. However, there are still barriers to the genetic analysis of diverse *Bacteroidales* strains, limiting our ability to study important host and community phenotypes identified in these strains. Here, we have overcome many of these obstacles by constructing a series of vectors that allow easy genetic manipulation in diverse gut *Bacteroides* and *Parabacteroides* strains. These constructs fill a critical need and allow streamlined allelic replacement in diverse gut *Bacteroidales*, including the growing number of multiantibiotic-resistant strains present in the modern-day human intestine.

## INTRODUCTION

The human gut microbiota is a diverse and dense microbial ecosystem that plays an essential role in health and development (1, 2). Large-scale metagenomic, metatranscriptomic, and metabolomic studies are revealing the molecular repertoire of this complex community (3, 4), yet functional genetic, phenotypic, and mechanistic studies lag far behind and are essential to understand the complex interactions within this microbial ecosystem and how these microbes interface with their host (3, 4).

The *Bacteroidales* are an order of Gram-negative bacteria that includes the abundant gut genera *Bacteroides*, *Parabacteroides*, and *Prevotella*, which collectively include more than 55 identified human gut species (5, 6). These bacteria display remarkable stability and substantial resilience to temporary perturbations, with many strains colonizing for decades (7–9). The majority of genetic and phenotypic studies of the gut *Bacteroidales* have analyzed only three strains: Bacteroides fragilis NCTC 9343, B. fragilis 638R, and B. thetaiotaomicron VPI 5482 (10, 11). However, *Bacteroidales* species have remarkable genome plasticity, with extensive within-species genetic diversity and metabolic abilities and large pangenomes (6, 12, 13). Many of the regions involved in the synthesis of outer surface and secreted molecules that are likely to interface with other microbes or the host are in heterogeneous regions of a species (12, 14–20). This heterogeneity highlights the need for functional genetic studies of diverse human isolates.

*Bacteroides* species are intrinsically resistant to many of the antibiotics commonly used in the laboratory and now display increasing resistance to many antibiotics used in clinical settings (21–28). Due to pervasive horizontal gene transfer of antibiotic resistance genes, many *Bacteroides* and *Parabacteroides* isolates from American and European subjects now display resistance to tetracycline and erythromycin, the two commonly used antibiotics for genetic selection (25, 29–31). Chloramphenicol and cefoxitin resistance genes have been used to select for replicative plasmids in B. fragilis, B. vulgatus, and B. thetaiotaomicron strains (29, 32, 33) and sometimes function for chromosomal integrations in select genetic backgrounds (29, 31, 34–36). However, cefoxitin and carbapenems are clinically relevant antibiotics and should ideally be avoided in the laboratory to prevent the spread of resistance genes (37).

Construction of markerless gene knockouts or other types of mutations in bacteria can be carried out through lambda red recombineering or CRISPR-Cas9-based systems (38–40). However, these methods often require strain-specific modifications; rely on efficient transformation, which is often impossible or very inefficient in *Bacteroidales*; and may lead to off-target mutations (38–42). Allelic replacement in *Bacteroides* is a two-step process. First, a suicide vector is transferred via conjugation and integrated into the chromosome via homology-based recombination of regions flanking the target deletion and selected via a gain-of-function phenotype, typically antibiotic resistance. This limits the tractable strains to those sensitive to erythromycin or tetracycline and therefore excludes a large number of isolates. Genomes that undergo double-crossover events result in the excision of the vector and typically are identified by one of three different methods. The first is replica plating, which has the advantages that there is no need for a genetic mutation to first be made in the background strain and that the resulting strains therefore have no additional underlying genetic mutation. The disadvantage is that this is a more labor-intensive and slow process, requiring the analysis of thousands of colonies. A second method uses counterselection where a genetic mutation of *thyA*, encoding thymidylate synthetase, is made in the background strain (64). *thyA* is placed on the suicide vector, and double-crossover resolvents are selected by plating on trimethoprim, which kills *thyA*-positive cells. A third method is similar to the *thyA* procedure but requires the creation of a background mutant strain of *tdk*, encoding thymidine synthase, with the selection of resolvents by plating on 5-fluoro-2′-deoxyuridine (50). Other counterselection systems readily used in proteobacteria, such as *sacB* and *rpsL* (42), do not function in *Bacteroides*. Recently, a new counterselection marker was implemented based on an allele of the phenylalanyl-tRNA synthetase (pheS*), lethal in the presence of *p*-chloro-phenylalanine (*p*-Cl-Phe) (43). This system functions in some strains but not others, requires growth on minimal medium, and often results in significant background growth.

Here, we created a new vector family for genetic modifications in gut *Bacteroidales*. These vectors allow easy allelic replacement without first making a mutant background strain for counterselection. In addition, we created vectors where the gain of function is the ability to utilize a polysaccharide rather than antibiotic resistance, greatly increasing the range of strains that can be genetically manipulated to include multiresistant strains. We provide numerous examples of these vectors functioning in diverse *Bacteroidales* species, regardless of the antibiotic resistance profile.

## RESULTS AND DISCUSSION

### An inulin selection cassette for use in antibiotic-resistant *Bacteroidales* strains.

**FIG 1:**
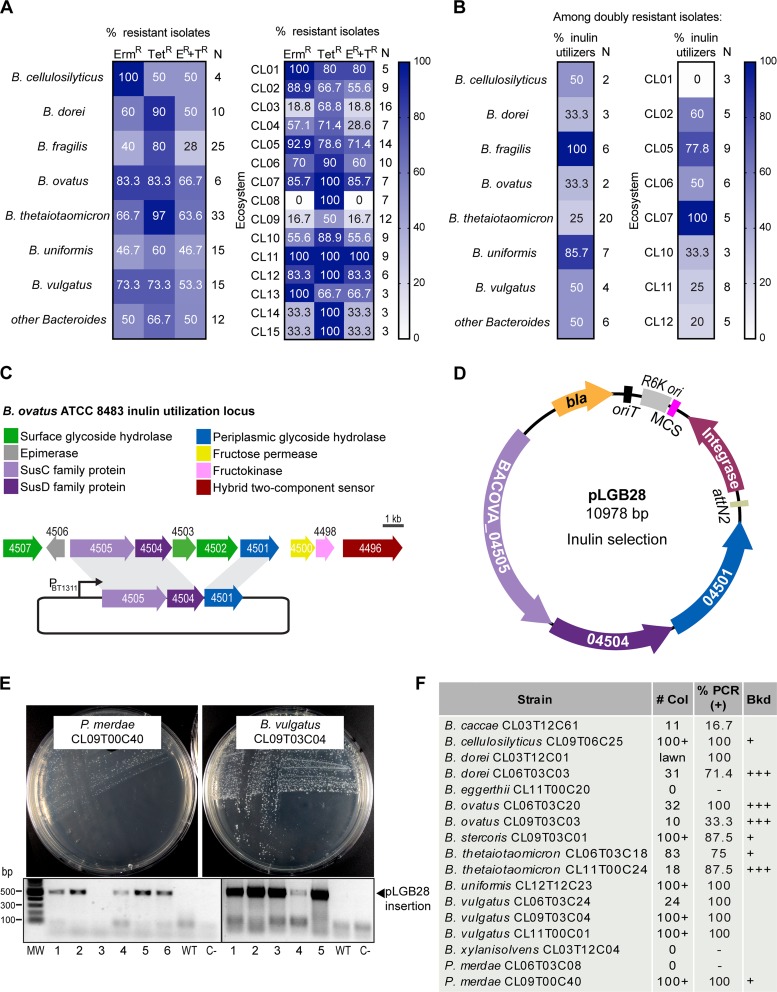
A minimal inulin utilization locus can be used for selection of integrants in many *Bacteroidales* species. (A) Percentage of tetracycline- and erythromycin-resistant *Bacteroides* strains from the Comstock laboratory ecosystems strain collection. Numbers in the right column (N) indicate the number of strains tested. (B) Percentage of inulin utilizers from the doubly resistant (Tet^r^ Erm^r^) *Bacteroides* strains. Numbers in the right column (N) indicate the number of strains tested. Ecosystems with fewer than three doubly resistant strains were excluded. (C) Inulin utilization locus of *B. ovatus* ATCC 8483. P_BT1311_ is the strong constitutive promoter from the *BT_1311* sigma factor of B. thetaiotaomicron VPI 5482 used to drive the expression of the three-gene inulin utilization locus. (D) Integration vector pLGB28 integrates at chromosomal *attBT2* sites and allows for inulin utilization selection. *bla*, β-lactamase gene conferring ampicillin and carbenicillin resistance in E. coli S17; *oriT*, RP4 origin of transfer; RK6, replication origin in E. coli λ*pir*; MCS, multiple-cloning site; Integrase, IntN2 from NBU2; *attN2*, * *recognition and integration sequence. (E) Growth of the indicated strains on M9S-inulin plates to select for integration of pLGB28. Ethidium bromide-stained agarose gels show PCR amplification of the 500-bp fragment corresponding to integration of pLGB28 at *attBT2*. Six clones are shown per strain. WT, wild type; C−, no-DNA control; MW, molecular weight marker (Quick-Load Purple 1-kb Plus DNA ladder; NEB). (F) Results of the conjugation of pLGB28 into 17 different doubly resistant (Tet^r^ Erm^r^), inulin-nonutilizing *Bacteroides* and *Parabacteroides* strains from panel B. # Col, number of colonies obtained per 13.75 ml of mating culture mix; %PCR (+), percentage of clones that were PCR positive as described above for panel E; Bkd, amount of background growth in the inulin selection plate; +, slight hazy background with distinct colonies (as observed in panel E, left); +++, strong background growth (as observed in Fig. 3B, left).

Analyses of recent human fecal isolates of *Bacteroidales* with interesting phenotypes revealed that many are resistant to both tetracycline and erythromycin (60, 65) and therefore are not amenable to genetic manipulation using available genetic tools for these bacteria. We evaluated tetracycline and erythromycin resistance among 120 *Bacteroides* strains from our collection isolated between 2009 and 2011 from the stool of 15 healthy U.S.-resident adults with no history of gastrointestinal diseases (Fig. 1A) (18). For each subject/ecosystem (named CL01 to CL15), the stated numbers of *Bacteroides* isolates analyzed are listed (Fig. 1A) (18). On average, 59% of the isolates from these combined ecosystems are resistant to erythromycin, and 74% are resistant to tetracycline, while 51% are doubly resistant. In particular, 63% of B. ovatus and 67% of B. thetaiotaomicron isolates from these ecosystems are doubly resistant. The incidence of resistance varies widely depending on the ecosystem (Fig. 1A), with isolates from ecosystems CL03, CL08, and CL14 displaying low levels of double resistance, while ecosystems CL01, CL05, CL07, CL11, and CL12 are almost entirely composed of doubly resistant isolates. These results are consistent with other reports of widespread resistance to tetracycline and erythromycin in recent *Bacteroides* and *Parabacteroides* isolates (22, 23, 31).

To overcome the genetic intractability of these strains, we designed a nonantibiotic selection marker that confers the ability to utilize inulin as a carbon source. To test if inulin selection would be useful in the doubly antibiotic-resistant isolates, we screened 57 of these strains for growth on defined, minimal inulin medium (M9S-inulin) (Fig. 1B) and observed that 28 (49%) are inulin utilizers. Excluding B. fragilis and B. uniformis, the majority of doubly resistant isolates of other *Bacteroides* species (72%) lack the ability to grow on inulin and are therefore theoretically amenable to inulin selection.

The choice of conferring inulin utilization for gain-of-function selection of cointegrates is based on the fact that inulin is a simple linear polysaccharide ([β1,2] fructose polymer) that does not require surface digestion (20, 44). Therefore, the inulin utilization locus is smaller than most polysaccharide utilization loci (PULs), which typically span 20 to 60 kb (10, 45, 46), and includes several dispensable genes (Fig. 1C) (20, 44, 47). The absence of extracellular digestion prevents background growth of nontransconjugant cells (44). We constructed a three-gene inulin utilization cassette from the inulin locus of *B. ovatus* ATCC 8483. It comprises genes encoding the outer membrane SusC-like TonB-dependent inulin transporter (*BACOVA_04505*), its associated inulin-binding surface lipoprotein (SusD-like; *BACOVA_04504*), and the periplasmic glycoside hydrolase (*BACOVA_04501*) (44, 48) (Fig. 1C). The three genes were placed under the control of a strong constitutive promoter from the B. thetaiotaomicron VPI 5482 housekeeping sigma factor P_BT1311_ (49). This inulin selection cassette (6.7 kb) was placed into pNBU2 (50), replacing the erythromycin cassette, to create pLGB28 (Fig. 1D). pNBU2 is a *pir*-dependent suicide vector which replicates in certain Escherichia coli strains but must integrate for maintenance in *Bacteroides* species. pNBU2 encodes an IntN2 tyrosine recombinase that catalyzes the integration of the vector at a chromosomal *attBT2* site (50, 51). Most *Bacteroides* strains have two *attBT2* sites, located at the 3′ end of the two tRNA^Ser^ genes (51). Double-integration events are not expected, as the integration of the plasmid disrupts the tRNA gene (51).

We conjugally transferred pLGB28 into 17 doubly resistant *Bacteroides* and *Parabacteroides* strains unable to utilize inulin as a sole carbon source. We obtained inulin-utilizing integrants for 14 of these strains from nine different species (Fig. 1E and F). Four strains displayed some background growth, which required restreaking of single colonies for isolation. As inulin selection is not bactericidal, we recommend that integrants always be restreaked for isolation. For the majority of strains, there were numerous integrants with little or no background growth. Notably, we easily obtained transconjugants in Parabacteroides merdae CL09T00C40 (*Pm*CL09), indicating that these protocols and vector series can be used in different families of *Bacteroidales*. Interestingly, both *attBT2* sites in *Pm*CL09 have two base pair differences relative to the *attBT2* sequence of pNBU2 and *Bacteroides* chromosomes (51), indicating some promiscuity for IntN2-mediated recombination. To verify integration at the *attBT2* sites, we designed PCR primers that anneal upstream of the recombination site (forward primer) and inside pNBU2 (reverse primer). Most integrates in the majority of strains had the insertion at one of the two tRNA^Ser^ loci. However, in *B. ovatus* CL09T03C03 and B. caccae CL03T12C61, PCR revealed that most inulin-utilizing clones did not have an insertion of the plasmid in tRNA^Ser^. Importantly, these data show that selection of integrants using inulin selection is a feasible alternative to antibiotic selection for most doubly resistant *Bacteroides* species.

### The aTC-inducible ssBfe1 is a highly effective counterselection system.

**FIG 2:**
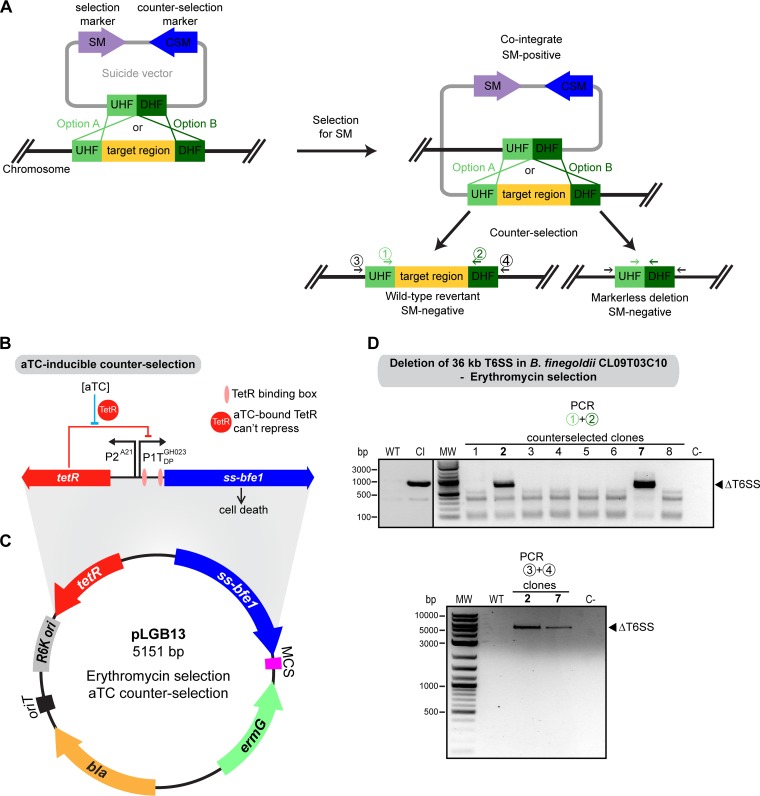
Bfe1-based counterselection induced by aTC is highly effective. (A) Diagram of the two-step allelic replacement process. UHF, upstream homology flank; DHF, downstream homology flank. (B) aTC-inducible ssBfe1 counterselection cassette (53). (C) Vector pLGB13. *bla*, OriT, RK6, and MCS are described in the Fig. 1D legend. *ermG*, erythromycin resistance gene. (D) PCR verification of the counterselected clones for deletion of the 36-kb T6SS region of *Bfi*CL09. WT, wild type; CI, cointegrate; C−, no-DNA control; MW, molecular weight marker. (Top) PCR using primers 1 and 2; (bottom) PCR using primers 3 and 4, as indicated in panel A.

Two-step allelic exchange (Fig. 2A) involves an intermediate cointegrate step where the suicide vector containing the genes for positive selection is integrated into the chromosome at the target site, followed by growth in nonselective medium and plating with selection for cells that have undergone the second recombination (cross-out) event. Selection of double-crossover resolvents using a lethal toxin encoded on the plasmid to kill cointegrates should be an effective counterselection system. Three essential features of such a counterselection system are the low frequency of toxin-resistant escapees, broad host range of toxicity, and tight regulation of the toxin (produced only during the counterselection step). The highly toxic effector Bfe1 from the B. fragilis 638R type VI secretion system (T6SS), which mediates antibacterial antagonism against a wide range of *Bacteroidales* species (52), was chosen for counterselection. Lim et al. showed that Bfe1 can be endogenously expressed in B. thetaiotaomicron VPI 5482 and kill this strain when targeted to the periplasm using an N-terminal localization signal sequence (ssBfe1) (53). When placed under the tightly regulated anhydrotetracycline (aTC)-inducible promoter developed for *Bacteroides* by Lim et al. (Fig. 2B), periplasmic Bfe1 expression leads to rapid cell death, with no evidence of escapees (53). Furthermore, although *Bacteroides* species have been shown to commonly harbor islands of immunity genes to multiple T6SS effectors (54), the *bfi1* (*BF638R_1987*) immunity gene to Bfe1 (52) is absent from these islands. tBLASTn searches of 724 available *Bacteroides* and *Parabacteroides* genomes revealed that only 35 B. fragilis strains have *bfi1*, all of them as part of a T6SS cluster containing *bfe1* (complete genomes and whole-genome shotgun contigs in GenBank, along with our in-house-sequenced strains).

As this system met all the criteria for successful counterselection, we made a counterselection suicide vector. Starting from the aTC-inducible pNBU2_erm-TetR-P1T_DP-GH023 integration vector (53), we removed the *attBT2* site and its cognate integrase gene, necessitating integration by homologous recombination. The *ssbfe1* cassette was added under the control of the aTC promoter. The multiple-cloning site for insertion of DNA for homology-based recombination was retained (pLGB13) (Fig. 2B and C).

To test pLGB13 for creating chromosomal deletions, we made a construct to delete the 36-kb GA1 T6SS locus (*HMPREF1057_01517* to *HMPREF1057_01551*) of B. finegoldii CL09T03C10 (*Bfi*CL09) (Fig. 2D). All colonies obtained after counterselection (on aTC selection plates) were erythromycin sensitive, indicating loss of the vector. PCR verification of eight clones indicated that six were wild-type revertants and that two had the desired 36-kb deletion (clones 2 and 7). To further verify this large deletion, we designed a second set of primers that anneal to the chromosome in the regions upstream and downstream of the flanking DNA cloned into pLGB13 (primers 3 and 4) (Fig. 2A). A PCR with these primers in clones 2 and 7 yielded the expected amplicon size (5.2 kb), whereas a cointegrate or a wild-type revertant would produce no amplicon (Fig. 2D). This pLGB13 counterselection system has now been successfully used in the laboratory to make deletions and allelic replacements in strains of B. uniformis, B. thetaiotaomicron, and B. vulgatus.

**FIG 3:**
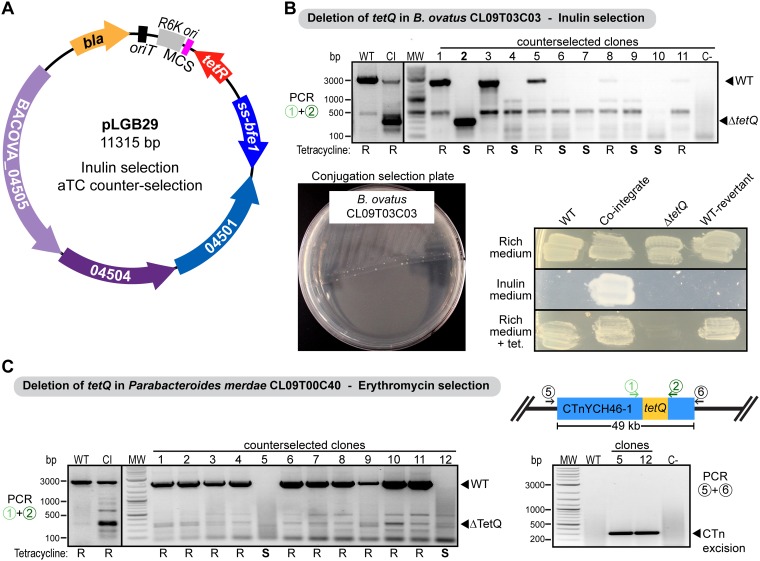
Deletion of the tetracycline resistance gene *tetQ*. (A) Allelic replacement vector pLGB29, using inulin selection for cointegrates. *bla*, *oriT*, RK6, and MCS are described in the Fig. 1D legend. (B) PCR verification and growth on plates of the counterselected clones for the *tetQ* deletion in *Bo*CL09. An inulin selection plate for the mating of *Bo*CL09T03C03, using homology-based integration of pLGB29 containing 1 kb of DNA on each side of the *tetQ* gene, is shown. S, sensitive; R, resistant. (C, left) Ethidium bromide-stained agarose gel showing PCR amplicons of the resolvent for the *tetQ* deletion in *Pm*CL09 using pLGB13 (erythromycin sensitive). (Right) PCR amplicon to verify excision of the whole CTnYCH46-1 (*HMPREF1078_01847* to *HMPREF1078_01893*). Primers anneal upstream and downstream of the 49-kb conjugative transposon. WT, wild type; CI, cointegrate. C−, no-DNA control; MW, molecular weight marker.

pLGB13 requires that the strain to be mutated is erythromycin sensitive. To extend this counterselection system to erythromycin-resistant strains, we replaced *ermG* of pLGB13 with the inulin utilization cassette, creating plasmid pLGB29 (Fig. 3A). As a proof of concept for inulin selection with homology-based recombination, we cloned into pLGB29 the 1-kb regions upstream and downstream of the *tetQ* tetracycline resistance gene of *B. ovatus* CL09T03C03 (*Bo*CL09) (*AA414_04155*). Cointegrates were obtained for *Bo*CL09 (Fig. 3B); however, there was background growth such that clones needed to be streaked onto inulin plates twice to obtain true cointegrates. We verified the cointegrates using PCR primers that anneal within the homology flanks (depicted in Fig. 2A as primer pair 1 and 2), such that amplification from the cointegrate should yield both the wild-type *tetQ* band (2.2 kb) as well as the shorter product representing the deletion (300 bp) (Fig. 3B).

**FIG 4:**
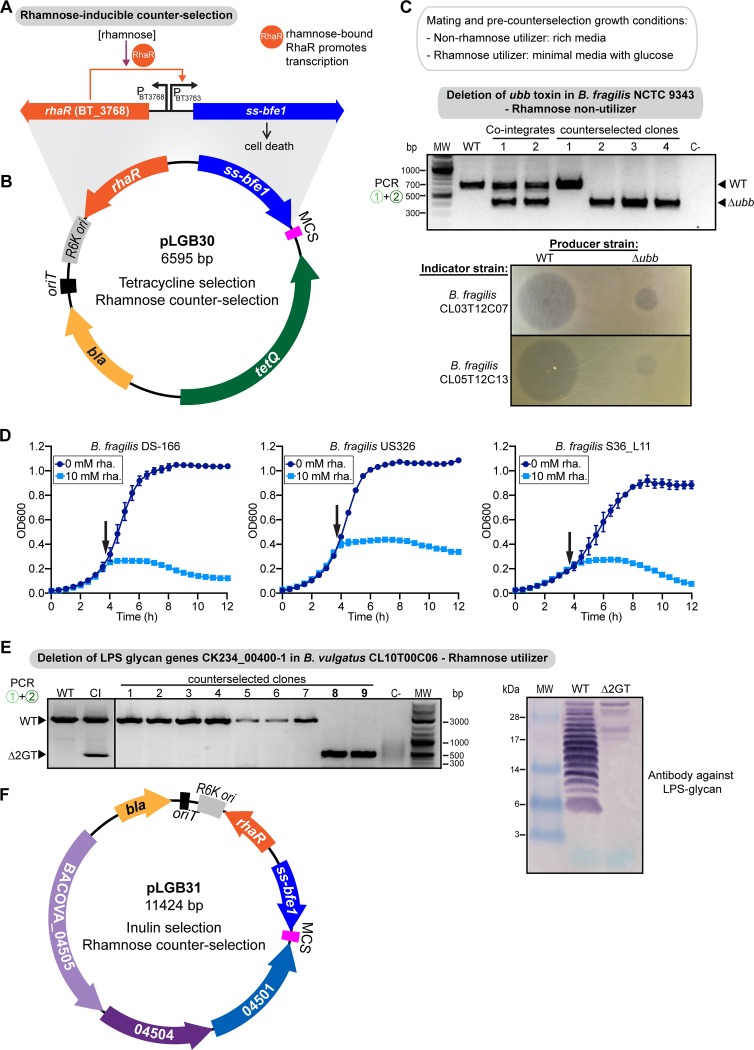
Bfe1-based counterselection induced by rhamnose. (A) Rhamnose-inducible ssBfe1 counterselection cassette. (B) Allelic replacement vector pLGB30 using tetracycline selection of cointegrates. *bla*, *oriT*, RK6, and MCS are described in the Fig. 1D legend. *tetQ*, tetracycline resistance gene. (C, top) PCR verification of the cointegrates and counterselected clones for the *ubb* deletion in *Bf*9343, using primers 1 and 2, as indicated in the Fig. 2A legend. WT, wild type; CI, cointegrate; C−, no-DNA control; MW, molecular weight marker. (Bottom) Overlay assays to test BfUbb inhibitory activity by wild-type or Δ*ubb Bf*9343, using the specified strains as indicators and producers. (D) Growth curves of the specified B. fragilis strains containing a chromosomally integrated rhamnose-inducible *ssbfe1*. Black arrows indicate the timing of rhamnose addition to the culture. The means and SEM from 3 biological replicates per treatment are plotted. (E, left) PCR verification of the counterselected clones for the deletion of *CK234_00400-1* in *Bv*CL10, using primers 1 and 2, as indicated in the Fig. 2A legend. (Right) Western immunoblotting of whole-cell lysates probed with antiserum raised to WT *Bv*CL10 adsorbed with the Δ*CK234_00400-1* mutant. (F) Allelic replacement vector pLGB31 using inulin selection. *bla*, *oriT*, RK6, and MCS are described in the Fig. 1D legend.

For comparison of inulin selection against traditional erythromycin selection, we cloned the same 1-kb regions upstream and downstream of *tetQ* into pLGB13 (erythromycin selection) to delete this gene in *Pm*CL09 (erythromycin sensitive; *HMPREF1078_01857*). Cointegrates of *Bo*CL09ΩpLGB29 (inulin) or *Pm*CL09ΩpLGB13 (erythromycin) were grown in nonselective broth and plated onto medium containing 40 ng/ml aTC. None of the aTC-selected colonies grew on either inulin (*Bo*CL09) or erythromycin (*Pm*CL09), indicating plasmid excision. However, PCR analysis and growth on plates containing tetracycline, whose resistance is encoded by the targeted deletion, indicated that most clones were wild-type revertants (Fig. 3B and C). In 1 of 30 resolvents tested for *Bo*CL09, we identified a mutant that produced the expected 300-bp PCR band corresponding to the *tetQ* deletion (clone 2) (Fig. 3B). Interestingly, five resolvents displayed no deletion band or the wild-type band (clones 4, 6, 7, 9, and 10) (Fig. 3B). Inspection of the genomic context of the *tetQ* gene in both strains revealed that it is part of a 49-kb conjugative transposon (CTn) with 99% sequence identity to CTnYCH46-1 that belongs to the CTn*341* family (55). The excision and transfer of CTn*341* (and CTnYCH46-1) are induced by tetracycline (and its derivative aTC), mediated through the two-component regulatory system RteAB (56). Therefore, selection using aTC induced the excision of the whole CTnYCH46-1 during counterselection. We observed a similar phenomenon in *Pm*CL09, where the majority of colonies after counterselection were wild-type revertants and two were CTnYCH46-1 excisions (clones 5 and 12) (Fig. 3C). We used PCR with primers annealing upstream and downstream of this 49-kb element and confirmed the excision of CTnYCH46-1 (*HMPREF1078_01847* to *HMPREF1078_01893*) in these clones (Fig. 3C). Therefore, although the aTC-ssBfe1 cassette is highly efficient for counterselection, it is not ideal for deletion of genes contained on tetracycline-inducible CTns. Since aTC induction also precludes the use of tetracycline as a selection marker, which would be desired in tetracycline-sensitive erythromycin-resistant strains, we devised a rhamnose-inducible alternative to be used in a tetracycline selection vector (pLGB30) (Fig. 4B) or an inulin selection vector (pLGB31) (Fig. 4F), described below. pLGB31 can also be used for allelic replacement of genes contained in the tetracycline-inducible CTn.

### The rhamnose-inducible ssBfe1 system.

Mimee et al. showed that the RhaR (BT_3768) transcriptional activator from B. thetaiotaomicron VPI 5482 can be used to drive robust activation of the *BT_3763* (*rhaK*) promoter (Fig. 4A) (57, 58). We placed ss*bfe1* under the control of this promoter cassette, creating suicide vectors pLGB30 (tetracycline selection) (Fig. 4B) and pLGB31 (inulin selection) (Fig. 4F). As some strains of *Bacteroidales* are able to grow with rhamnose as the sole carbon source and others are not, we analyzed whether this system would function in strains with each of these phenotypes. We first tested this system in B. fragilis NCTC 9343 (*Bf*9343) (Tet^s^), which does not have the ability to grow using rhamnose as the sole carbon source and whose genome does not harbor a described rhamnose utilization locus. Using pLGB30, we made a construct to delete the gene encoding the antimicrobial toxin BfUbb (BF9343_3779), which inhibits the growth of other B. fragilis strains (59). Despite the inability of *Bf*9343 to utilize rhamnose, the system drove the expression of *ssbfe1* to high-enough levels that only double-crossover resolvents grew on the rhamnose counterselection plates, and all these colonies lost pLGB30, as they were not tetracycline resistant. Therefore, *Bf*9343 cells have some ability to take up rhamnose, although they do not have a described RhaT permease (58). PCR analysis of four resolvents identified three with the correct *ubb* deletion (clones 2, 3, and 4) (Fig. 4C). We verified this deletion phenotypically by testing for killing activity using the spot agar overlay assay (Fig. 4C). Wild-type *Bf*9343 produced a large zone of growth inhibition for the two tested BfUbb-sensitive strains, whereas the *Bf*9343 Δ*ubb* strain lost this ability. A small inhibition zone was observed, corresponding to the size of the original bacterial spot, demonstrating that *Bf*9343 produces an additional antimicrobial toxin, as is typical of B. fragilis strains (59, 60). These analyses show that pLGB30 allows for easy creation of double-crossover mutants in erythromycin-resistant strains where tetracycline selection of cointegrates is necessary.

To determine if this vector can be widely used in non-rhamnose-utilizer strains, we carried out BLAST searches of all 724 available *Bacteroides* and *Parabacteroides* genomes. We used as queries the rhamnose symporter (*BT_3765*), rhamnulose-1-phosphate aldolase (*BT_3766*), and rhamnulose kinase (*BT_3763*) genes from B. thetaiotaomicron VPI 5482 (58). All *Bacteroides* and *Parabacteroides* genomes from all species had >90% identity hits, indicating that rhamnose utilization is a conserved trait. The exceptions were all 167 strains of B. fragilis. To establish if rhamnose induction of ssBfe1 would function for counterselection in other strains of B. fragilis, which homology-based searches suggest are also unable to utilize rhamnose, we made a pNBU2-derivative chromosomal integration vector carrying the RhaR-*ssbfe1* cassette. We integrated this construct into three B. fragilis strains and monitored their growth with or without added rhamnose (Fig. 4D). For all three strains, rhamnose addition led to a very rapid growth arrest, with no evidence of resumed growth up to 8 h after toxin induction. Therefore, even though B. fragilis strains are unable to utilize inulin as a sole carbon source, this rhamnose-inducible toxin system functions broadly in this species.

Next, we tested this counterselection system in a rhamnose-utilizing strain, B. vulgatus CL10T00C06 (*Bv*CL10). Unlike B. fragilis, *Bo*CL09 has a rhamnose permease as well as a rhamnose two-component signaling system, likely leading to induction of the *rhaK* promoter at much lower concentrations of rhamnose than for a rhamnose nonutilizer. Therefore, all steps prior to counterselection were performed using defined M9S-glucose medium, as small amounts of rhamnose in BHIS plates or supplemented basal medium (SBM) broth could induce premature expression of the toxin. We made a construct in pLGB30 to delete the genes *CK234_00400* and *CK234_00401* (*CK234_00400-1*). These genes encode glycosyltransferases involved in the synthesis of the lipopolysaccharide (LPS) glycan (15). Counterselection on M9S-rhamnose plates yielded single colonies, all tetracycline sensitive. PCR analysis demonstrated that of 10 resolvents analyzed, 2 were deletion mutants (clones 8 and 9) (Fig. 4E). We further verified the phenotype of this deletion mutant by Western immunoblotting of whole-cell lysates using an antiserum specific to the LPS glycan of *Bv*CL10 (15). The antibody reactivity to this glycan is lost in the Δ*CK234_00400-1* mutant (Fig. 4E), providing phenotypic confirmation of the deletion.

To further increase the breadth of strains that can be mutated and the genetic pool to include allelic replacement of genes in tetracycline-inducible CTns, we created pLGB31 (Fig. 4F). This vector is similar to pLGB29 in that cointegrates are selected by the acquisition of inulin utilization but with rhamnose as the inducer of *ssbfe1* for selection of double-crossover resolvents.

In summary, we have created a versatile family of vectors and protocols to greatly facilitate genetic manipulation of diverse *Bacteroides* and *Parabacteroides* strains. These vectors allow for allelic deletions and replacements in the increasingly abundant antibiotic-resistant strains that are currently not amenable to genetic manipulation with existing genetic tools. Routine mutant construction times are reduced from several weeks to less than 2 weeks, and the steps are much less labor-intensive than protocols without counterselection. In addition, there is no need to create a mutant background strain for counterselection. Bfe1 is a broad-range and potent toxin, and we have not encountered instances of spontaneous resistance mutations in any species. This is an easily modifiable system where the ssBfe1 toxin gene could be exchanged for a different GA3 T6SS toxin gene for genetic manipulation in B. fragilis strains, such as 638R, that encode the cognate immunity protein Bfi1. The versatility of two different inducers of Bfe1 increases the breadth of strains and genes that can be genetically manipulated.

## MATERIALS AND METHODS

### Media and growth conditions.

E. coli strains were grown aerobically at 37°C in LB medium, with 100 μg/ml carbenicillin added when indicated. *Bacteroides* and *Parabacteroides* strains were routinely grown at 37°C under anaerobic conditions on supplemented basal medium (SBM) (liquid cultures) (61) and brain heart infusion plates supplemented with 5 mg/liter hemin and 2.5 μg/liter vitamin K_1_ (BHIS). When necessary for selection, M9S plates were used, which are M9 minimal medium (62) supplemented with 50 mg/liter l-cysteine, 5 mg/liter hemin, 2.5 μg/liter vitamin K_1_, 2 mg/liter FeSO_4_ · 7H_2_O, 5 μg/liter vitamin B_12_, and 0.7% agarose. The carbon source for these plates was either 0.25% (wt/vol) glucose (M9S-glucose) or 0.4% (wt/vol) inulin (M9S-inulin) (inulin from chicory; Sigma-Aldrich). Antibiotics were used at the following concentrations, where appropriate: 5 μg/ml erythromycin, 200 μg/ml gentamicin, and 6 μg/ml tetracycline. Anhydrotetracycline (aTC) was added at 40 or 100 ng/ml, while l-(+)-rhamnose was used at 10 mM. For growth curves, bacteria from a culture grown overnight in broth were diluted 1:200 in fresh medium, allowed to enter exponential growth, and diluted again to an optical density at 600 nm (OD_600_) of 0.02. Growth curves were carried out in flat-bottom 96-well plates, with 200 μl per well, under anaerobic conditions at 37°C. OD_600_ values were measured every 30 min with an Eon high-performance microplate spectrophotometer (BioTek Instruments). A total of 10 mM rhamnose was added to the corresponding wells after 3 h 35 min of incubation. The means and standard errors of the means (SEM) from 3 biological replicates per treatment were plotted in Prism 8 for macOS (GraphPad Software).

### Plasmid construction.

Table S1 in the supplemental material summarizes the plasmids and construction methods used in this study. Phusion high-fidelity DNA polymerase or Q5 high-fidelity DNA polymerase was used for PCR cloning steps (New England Biolabs [NEB]), and all restriction endonucleases were high-fidelity restriction endonucleases from NEB. All plasmid assembly reactions were carried out using NEBuilder HiFi DNA assembly master mix (NEB). Oligonucleotides used for plasmid construction and PCR strain verification are listed in Table S2. Whole-plasmid sequencing was performed at the Massachusetts General Hospital CCIB DNA Core. The inulin utilization cassette causes a fitness defect in E. coli, leading to colonies that are smaller and more mucoid than the wild type and take 24 h to appear. These should be preferentially picked over the faster-appearing, nonmucoid colonies, which are likely to carry loss-of-function mutations. In order to avoid accumulation of mutations in the inulin plasmid, we recommend minimizing propagation steps and working on a Tn*10*-negative cloning strain.

10.1128/mBio.01762-19.1 TABLE S1 Plasmids and construction details. Download Table S1, PDF file, 0.04 MB. Copyright © 2019 García-Bayona and Comstock. 2019 García-Bayona and Comstock This content is distributed under the terms of the Creative Commons Attribution 4.0 International license.

10.1128/mBio.01762-19.2 TABLE S2 Primers used in this study. Download Table S2, PDF file, 0.03 MB. Copyright © 2019 García-Bayona and Comstock. 2019 García-Bayona and Comstock This content is distributed under the terms of the Creative Commons Attribution 4.0 International license.

### Conjugation and selection conditions.

Parental strains and strains constructed in this study are listed in Table S3. Plasmids were transformed into the donor E. coli strain S17-λ*pir* and conjugated into *Bacteroides* or *Parabacteroides* as described previously (31, 63), with some modifications. Briefly, donor and recipient strains were grown on liquid medium, 25 ml and 2.5 ml, respectively. When the recipient strain reached an OD_600_ of between 0.1 and 0.2 (0.05 for B. fragilis) and the donor strain reached an OD_600_ of 0.2 to 0.6, both strains were mixed and pelleted by centrifugation at 9,000 × *g* for 10 min. The pellet was resuspended in 100 μl SBM, spotted directly in the center of a prewarmed BHIS plate, and incubated at 37°C aerobically for 15 to 18 h. The bacteria from the mating spot were streaked onto the appropriate selection plates (BHIS plus erythromycin, BHIS plus tetracycline, or M9S-inulin) containing gentamicin (one plate had half the mating spot, another plate had a quarter, and the last quarter of the mating spot was streaked for isolation in the third plate). For rhamnose counterselection using pLGB30 for rhamnose-utilizing strains, cointegrates were selected using M9S-glucose plates with gentamicin and tetracycline. Plates were incubated anaerobically. Colonies were picked after 2 to 3 days (BHIS and M9S-glucose) or 3 to 4 days (M9S-inulin) and restreaked for isolation. PCRs for strain verification were performed using Phusion DNA polymerase with the oligonucleotides listed in Table S2.

10.1128/mBio.01762-19.3 TABLE S3 *Bacteroidales* strains used or created in this study. Download Table S3, PDF file, 0.04 MB. Copyright © 2019 García-Bayona and Comstock. 2019 García-Bayona and Comstock This content is distributed under the terms of the Creative Commons Attribution 4.0 International license.

### Counterselection conditions.

After confirmation of cointegrates via PCR (Table S2), each strain was grown overnight in 6 ml SBM, diluted 1:100 in fresh SBM, and incubated for 6 to 8 h. Aliquots of 50, 5, 1, and 0.2 μl were plated onto BHIS plates containing 40 or 100 ng/ml aTC or 10 mM rhamnose, depending on the selection method. We have found that cointegrates can be grown from plates in nonselective medium for as little as an hour before plating for counterselection, eliminating the need for culture overnight. For rhamnose counterselection in rhamnose-utilizing strains, bacteria were grown in M9S-glucose broth and plated onto M9S-rhamnose (10 mM). After 2 to 4 days, single colonies were restreaked and analyzed by PCR to confirm the loss of the selection marker (Table S2).

### Agar overlay assays.

Agar spot overlay assays were carried out as previously described (30). Briefly, 5 μl of the toxin-producing strain or mutant was spotted onto plates and incubated overnight anaerobically. Cells were removed, and the plates were exposed to chloroform vapor to kill remaining bacteria. The overlay strain was grown to exponential phase, added to 4 ml warm BHIS with 0.8% agar, and poured on top of the chloroform-treated plate. Plates were incubated overnight anaerobically before imaging.

### Western blot analyses.

Western immunoblot analyses of exponential-phase cell lysates were performed as described previously, using antiserum prepared in rabbits to whole-cell B. vulgatus CL10T00C06, and subjected to antibody adsorption to B. vulgatus CL10T00C06 Δ*CK234_00400-1* (15).

### Data availability.

The vectors created in this study can be acquired from the Addgene repository (ID numbers 126617 to 126621; https://www.addgene.org/browse/article/28203359/).
