# Supplementary material for: Streamlined Genetic Manipulation of Diverse Bacteroides and Parabacteroides Isolates from the Human Gut Microbiota
Source: mBio. 2019 Aug 13;10(4):e01762-19. doi: 10.1128/mBio.01762-19 (PMC6692515; doi:10.1128/mBio.01762-19)
Supplement: TABLE S1 [file mBio.01762-19-st001.pdf]

Table S1. Plasmids and construction details

Name	Description	Construction details	Reference/Source
p*NBU2-bla-ermG*b	Integration at chromosomal *attBT2* site present in most *Bacteroides* and *Parabacteroides* isolates, erythromycin resistance		Koropatkin et al., 2008
p*NBU2_erm*-TetR-P1T_DP-GH023	Integration at *attBT2* site, cassette with P1T_DP aTC-inducible promoter (RBS GH023) and constitutively-driven TetR repressor		Lim et al., 2017
pLGB13	Suicide vector for allelic exchange. Erythromycin resistance cassette for co-integrate selection, aTC-inducible expression of ssBfe1 toxin for counter-selection	1) Chemically synthesized cassette (Genscript) containing a periplasmic localization signal (first 63 bp of *BT_4676* from *B. thetaiotaomicron* VPI-5482) fused to the *bfe1* gene (BF638R_1988) of *B. fragilis* 638R. (Lim et al., 2017)<br>2) Using NEBuilder assembly, this cassette was cloned into the NcoI site of *pNBU2_erm*-TetR-P1T_DP-GH023.<br>3) The resulting plasmid was used as template for PCR using oLGB1 and oLGB2, to remove the *attN2* site and the interase gene, and the resulting product was assembled using NEBuilder.	This study
pLGB18	Construct for deletion of the GA1 T6SS (*HMPREF1057_01516* to *HMPREF1057_01551*) in *B. finegoldii* CL09T03C10	NEBuilder assembly reaction to fuse:<br>a) pLGB13 cut with PstI and EcoRV<br>b) 2.6 kb flank upstream of *HMPREF1057_01516*, amplified using oLGB29 + oLGB30<br>c) 2.5 kb flank downstream of *HMPREF1057_01551*, amplified using oLGB31 + oLGB32	This study
pLGB28	Integration at chromosomal *attB2* site present in most *Bacteroides* and *Parabacteroides* isolates, inulin utilization cassette for positive selection	NEBuilder assembly reaction to fuse:<br>a) pNBU2 backbone excluding *ermG* cassette, amplified with oLGB78 and oLGB105<br>b) PBT1311 promoter and RBS, amplified from pMM553 (Mimee et al., 2015) using oLGB106 and oLGB107<br>c) *BACOVA_04504* and *BACOVA_04505* from *B. ovatus* ATCC 8483, amplified using oLGB104 and oLGB91<br>d) *BACOVA_04501* from *B. ovatus* ATCC 8483, amplified using oLGB85+86	This study

Name	Description	Construction details	Reference/Source
pLGB29	Suicide vector for allelic exchange. Inulin utiization cassette for co-integrate selection, aTC-inducible expression of ssBfe1 toxin for counter-selection	NEBuilder assembly reaction to fuse:<br>a) pLGB28 backbone excluding the *attN2* site and integrase gene, amplified with oLGB118 and oLGB119<br>b) counter-selection cassette, amplified from pLGB13 using oLGB116 and oLGB117	This study
pLGB30	Suicide vector for allelic exchange. Tetracycline resistance cassette for co-integrate selection, rhamnose-inducible expression of ssBfe1 toxin for counter-selection	NEBuilder assembly reaction to fuse:<br>a) rhamnose promoter, amplified from gene *BT_3763* from *B. thetaiotaomicron* VPI-5482, using oLGB108 and oLGB109<br>b) ss-*bfe1* gene and MCS, amplified from pLGB13, using oLGB110 and oLGB120<br>c) tetracycline resistance casette, amplified from vector pYT646b (Tang and Malamy, 2000), using oLGB121 and oLGB122<br>d) origin of transfer and replication, amplified from pLGB13 using oLGB111 and oLGB123<br>e) RhaR cassette (*BT_3768*), amplified from *B.thetaiotaomicron* VPI-5482, using oLGB112 and oLGB113	This study
pLGB31	Suicide vector for allelic exchange. Inulin utiization cassette for co-integrate selection, rhamnose-inducible expression of ssBfe1 toxin for counter-selection	NEBuilder assembly reaction to fuse:<br>a) ss-*bfe1* gene and MCS, amplified from pLGB13, using oLGB110 and oLGB152<br>b) rhamnose-inducible promoter and RhaR transcription factor, amplified from pLGB30 using oLGB109 and oLGB112<br>c) vector backbone (oriT/V, *bla* gene, inulin selection cassette) amplified from pLGB29 using oLGB86 and oLGB111	This study
pLGB32	Construct for deletion of *tetQ* in *B. ovatus* CL09T03C03 (*AA414_04155*)	NEBuilder assembly reaction to fuse:<br>a) pLGB29 cut with PstI and NotI<br>b) 1 kb flank upstream of *tetQ*, amplified using oLGB124 + oLGB125<br>c) 1 kb flank downstream of *tetQ*, amplified using oLGB126 + oLGB127	This study
pLGB33	Construct for deletion of *tetQ* in *P. merdae* CL09T00C40 (*HMPREF1078_01857*)	NEBuilder assembly reaction to fuse:<br>a) pLGB13 cut with PstI and EcoRV<br>b) 1 kb flank upstream of *tetQ*, amplified using oLGB124 + oLGB125<br>c) 1 kb flank downstream of *tetQ*, amplified using oLGB126 + oLGB153	This study

Name	Description	Construction details	Reference/Source
pLGB34	Construct for deletion of *ubb* in *B. fragilis* NCTC 9343 (*BF9343_3779*)	NEBuilder assembly reaction to fuse:<br>a) pLGB30 cut with PstI and SalI<br>b) 2.5 kb flank upstream of *ubb*, amplified using oLGB141 + oLGB142<br>c) 2.5 kb flank downstream of *ubb*, amplified using oLGB143 + oLGB144	This study
pLGB35	Construct for deletion of *CK234_00400-00401* in *B. vulgatus* CL10T00C06	NEBuilder assembly reaction to fuse:<br>a) pLGB30 cut with PstI and SalI<br>b) 1.7 kb and 1.8 kb flanks upstream and downstream of *CK234_00400-00401*, amplified from *Bv* CL10Δ2GT (McEneany et al., 2018) using oLGB147 + oLGB148	This study
pLGB37	Integration at chromosomal *attB2* site present in most *Bacteroides* and *Parabacteroides* isolates, erythromycin resistance cassette for positive selection, rhamnose-inducible expression of ssBfe1 toxin	NEBuilder assembly reaction to fuse:<br>a) vector backbone (oriT/V, *bla*, *ermG*) amplified from pNBU2 using oLGB167+168<br>b) rhamnose-inducible ss-*bfe1*, amplified from pLGB30 using oLGB165 and oLGB166	This study

References

Koropatkin,N.M., Martens,E.C., Gordon,J.I. and Smith,T.J. (2008) Starch Catabolism by a Prominent Human Gut Symbiont Is Directed by the Recognition of Amylose Helices. *Structure*, **16**, 1105–1115.

Lim,B., Zimmermann,M., Barry,N.A. and Goodman,A.L. (2017) Engineered Regulatory Systems Modulate Gene Expression of Human Commensals in the Gut. *Cell*, 169, 547-558.e15

McEneany,V.L., Coyne,M.J., Chatzidaki-Livanis,M. and Comstock,L.E. (2018) Acquisition of MACPF domain-encoding genes is the main contributor to LPS glycan diversity in gut Bacteroides species. The *ISME Journal*, 12, 2919–2928

Mimee,M., Tucker,A.C., Voigt,C.A. and Lu,T.K. (2015) Programming a Human Commensal Bacterium, *Bacteroides thetaiotaomicron*, to Sense and Respond to Stimuli in the Murine Gut Microbiota. *Cell Systems*, 1, 62–71.

Tang YP, Malamy MH. (2000) Isolation of Bacteroides fragilis mutants with in vivo growth defects by using Tn4400’, a modified Tn4400 transposition system, and a new screening method. *Infect Immun* 68:415– 419.
