# Supplementary material for: Streamlined Genetic Manipulation of Diverse Bacteroides and Parabacteroides Isolates from the Human Gut Microbiota
Source: mBio. 2019 Aug 13;10(4):e01762-19. doi: 10.1128/mBio.01762-19 (PMC6692515; doi:10.1128/mBio.01762-19)
Supplement: TABLE S2 [file mBio.01762-19-st002.pdf]

Table S2. Primers

Name	Sequence	Use
oLGB1	TGTATGAATCCAACAGTTGCATGTGGCCTATTGT	pLGB13 construction
oLGB2	ACATGCAACTGTTGGATTCATACAAGCGGTCG	pLGB13 construction
oLGB29	ATCCCCCGGGCTGCATGGCATAGCTACCGGTGGTAC	pLGB18 construction
oLGB30	TGCCTTTAATTCTGAAAGGGATTTCCTGACCGTTC	pLGB18 construction
oLGB31	GGAAATCCCTTTCAGAATTAAAGGCAACCGTGTG	pLGB18 construction
oLGB32	GGTATCGATAAGCTTGATTGGTCTTTCATCGCGTATCC	pLGB18 construction
oLGB33	GTTTCCGGTTGCGATAAGAG	T6SS deletion PCR verification in *Bfi*CL09
oLGB34	ATTTGGCGTTTCCTGTCAAC	T6SS deletion PCR verification in *Bfi*CL09
oLGB43	CGCATCATCAGAACGGTAAGTC	T6SS deletion PCR verification in *Bfi*CL09
oLGB78	ACAATAGGCCACATGCAACTG	pLGB28 construction
oLGB85	GTTTAACTAGCTCTAATACCATCTGACATGAA	pLGB28 construction
oLGB86	AGTTGCATGTGGCCTATTGTTTATGGCTTCAACTTATGAAC	pLGB28, pLGB31 construction
oLGB91	TGGTATTAGAGCTAGTTAAACTTACCGTATCCGGGA	pLGB28 construction
oLGB104	CATATGGCACTAATGCTTAGTATTATGAAGAACAAAAAACT	pLGB28 construction
oLGB105	CTTCTTCCAGATCACTCTTTAAGAGAAACTCTTCGGG	pLGB28 construction
oLGB106	TCTCTTAAAGAGTGATCTGGAAGAAGCAATGAAAG	pLGB28 construction
oLGB107	CATAATACTAAGCATTAGTGCCATATGTTAAAAACAGATTTGG	pLGB28 construction
oLGB108	CGGATGCTTATTATTTCGGAGAAAACATGCATAAATC	pLGB30 construction
oLGB109	ATTTTCTTCATGGTATTTTGTCTATATATTTAAGGTATCCATTTAC	pLGB30, pLGB31 construction
oLGB110	GACAAAATACCATGAAGAAAATTTTATCTTTGCTTGTGATG	pLGB30, pLGB31 construction
oLGB111	GGGAAGAATAAGGTGGAGGGGAATTCCCATG	pLGB30, pLGB31 construction
oLGB112	TCCCCTCCACCTTATTCTTCCCGACCGAACG	pLGB30, pLGB31 construction
oLGB113	TCTCCGAAATAATAAGCATCCGGAGGATCTG	pLGB30 construction
oLGB116	GAATGGATAGATCATAATGCTAATCTTACACCG	pLGB29 construction
oLGB117	ACCGCTTGTATCTTAAGACCCACTTTCACATTTAAG	pLGB29 construction
oLGB118	TGGGTCTTAAGATACAAGCGGTCGGTTCGGA	pLGB29 construction
oLGB119	TAGCATTATGATCTATCCATTCAGTTTGATTTCTCAGG	pLGB29 construction
oLGB120	ACTTATCTCCAAACAATAGGCCACATGCAACTGT	pLGB30 construction
oLGB121	GGCCTATTGTTTGGAGATAAGTATATATCCCAACTGTATTGC	pLGB30 construction
oLGB122	AAGAGAGAAACGTACTTACCAGTTGAACCTACGTTTCC	pLGB30 construction
oLGB123	CTGGTAAGTACGTTTCTCTCTTTAAGAGAAACTCTTCGGGATG	pLGB30 construction
oLGB124	GCTTGATATCGAATTCCTGCACATCATCGACCAGTACAAGGC	pLGB32, pLGB33 construction
oLGB125	ACCGCTCCATTTGGATTAAGCAATAATATACTACAGTAGATGC	pLGB32, pLGB33 construction
oLGB126	TATTGCTTAATCCAAATGGAGCGGTCAGGAAATT	pLGB32, pLGB33 construction
oLGB127	CCTCCACCGCGGTGGCAGCATATCCCGCATACGG	pLGB32 construction
oLGB128	GGTGCGTTTCGACAATGC	*tetQ* deletion PCR verification in *Bo*CL09 and *Pm*CL09
oLGB129	ATACGCCGTGCATGGTAATG	*tetQ* deletion PCR verification in *Bo*CL09 and *Pm*CL09
oLGB137	CGTTGTACTGCGAGGTACC	pLGB28 insertion PCR verification
oLGB138	GGTGGAAACGAGGAACAAGG	pLGB28 insertion PCR verification

Name	Sequence	Use
oLGB139	CGTTGTACCGCGAGGTACC	pLGB28 insertion PCR verification
oLGB141	GTGGATCCCCCGGGCTGCACTATTATACGCAAATCAACAGAACG	pLGB34 construction
oLGB142	CTCTTATGCAAATTTCAAAAGTACTTGTTTAATAAATCTCATTTG	pLGB34 construction
oLGB143	AACAAGTACTTTTGAAATTTGCATAAGAGATGTCGACTG	pLGB34 construction
oLGB144	CTGGAAGATAGGCAATTAGACTTTGAAGTCGATCCTGCC	pLGB34 construction
oLGB145	TCAACAGACCCTTCTTAAGGTA	*ubb* deletion PCR verification in *Bf* 9343
oLGB146	CTGTTAATACCGTGTCTGTATGATAC	*ubb* deletion PCR verification in *Bf* 9343
oLGB147	GTGGATCCCCCGGGCTGCAATGACTTCCAGGCATACAACC	pLGB35 construction
oLGB148	CTGGAAGATAGGCAATTAGTGTATCCAGCTGTCTGCAGAG	pLGB35 construction
oLGB149	CTAGAGGCTGAATATGAACGTG	*CK234_00400-00401* deletion PCR verification in *Bv* CL10
oLGB150	GTTCCGAACTTTCTAGTGAGC	*CK234_00400-00401* deletion PCR verification in *Bv* CL10
oLGB152	ATAAACAATAGGCCACATGCAACTGTTGGATTCATACAA	pLGB31 construction
oLGB153	GGTATCGATAAGCTTGATAGCATATCCCGCATACGG	pLGB33 construction
oLGB154	CCGTTGAACTGAGAGGTTCC	pLGB28 insertion PCR verification
oLGB155	CGTTGTGCTGCGAGGCAC	pLGB28 insertion PCR verification
oLGB160	ATTGACATACCTGTCCGTCGTC	CTn341 excision PCR verification in *Pm* CL09
oLGB161	GCATATATTGATGGTCGCCATCATC	CTn341 excision PCR verification in *Pm* CL09
oLGB164	CCCGTTTCCATTTATAGCCCTC	T6SS deletion PCR verification in *Bfi* CL09
oLGB165	CCTCCACCGCGGTTTATTCTTCCCGACCGAACGAG	pLGB37 construction
oLGB166	AGAGCGGCCTCATAATGCTAATCTTACACCGTCCC	pLGB37 construction
oLGB167	TAGCATTATGAGGCCGCTCTAGAACTAGTGGATCC	pLGB37 construction
oLGB168	TCGGGAAGAATAAACCGCGGTGGAGGGGAATTC	pLGB37 construction
oLGB169	ACGGCTGACATGGGAATTCC	pLGB37 insertion PCR verification
oLGB170	GCTTGATATCGAATTCCTGCAGC	pLGB37 insertion PCR verification
