# Supplementary material for: Streamlined Genetic Manipulation of Diverse Bacteroides and Parabacteroides Isolates from the Human Gut Microbiota
Source: mBio. 2019 Aug 13;10(4):e01762-19. doi: 10.1128/mBio.01762-19 (PMC6692515; doi:10.1128/mBio.01762-19)
Supplement: TABLE S3 [file mBio.01762-19-st003.pdf]

Table S3. Bacteroidales strains used or created in this study

Name	Genotype	Construction details	Reference/Source
B. caccae CL03T12C61	wild-type	N/A	Zitomersky *et al*., 2011
B. cellulosilyticus CL09T06C25	wild-type	N/A	Zitomersky *et al*., 2011
B. dorei CL03T12C01	wild-type	N/A	Zitomersky *et al*., 2011
B. dorei CL06T03C031	wild-type	N/A	Zitomersky *et al*., 2011
B. eggerthii CL11T00C20	wild-type	N/A	Zitomersky *et al*., 2011
B. finegoldii CL09T03C10	wild-type	N/A	Zitomersky *et al*., 2011
B. fragilis CL03T12C07	wild-type	N/A	Zitomersky *et al*., 2011
B. fragilis CL05T12C13	wild-type	N/A	Zitomersky *et al*., 2011
B. ovatus CL06T03C20	wild-type	N/A	Zitomersky et al., 2011
B. ovatus CL09T03C03	wild-type	N/A	Zitomersky *et al*., 2011
B. stercoris CL09T03C01	wild-type	N/A	Zitomersky *et al*., 2011
B. thetaiotaomicron CL06T03C18	wild-type	N/A	Zitomersky *et al*., 2011
B. thetaiotaomicron CL11T00C24	wild-type	N/A	Zitomersky *et al*., 2011
B. uniformis CL12T12C23	wild-type	N/A	Zitomersky *et al*., 2011
B. vulgatus CL06T03C24	wild-type	N/A	Zitomersky *et al*., 2011
B. vulgatus CL09T03C04	wild-type	N/A	Zitomersky *et al*., 2011
B. vulgatus CL10T00C06	wild-type	N/A	Zitomersky *et al*., 2011
B. vulgatus CL11T00C01	wild-type	N/A	Zitomersky *et al*., 2011
B. xylanisolvens CL03T12C04	wild-type	N/A	Zitomersky *et al*., 2011
P. merdae CL06T03C08	wild-type	N/A	Zitomersky *et al*., 2011
P. merdae CL09T00C40	wild-type	N/A	Zitomersky *et al*., 2011
B. fragilis NCTC 9343	wild-type	N/A	ATCC. Cato & Johnson, 1976
*Bca*CL03ΩpLGB28	*B. caccae* CL03T12C61 with pLGB28 integrated at *attBT2* site	Conjugal transfer of pLGB28 into B. caccae CL03T12C61, PCR confirmation using oLGB138 and oLGB139	This study
*Bce*CL09ΩpLGB28	*B. cellulosilyticus* CL09T06C25 with pLGB28 integrated at *attBT2* site	Conjugal transfer of pLGB28 into B. cellulosilyticus CL09T06C25, PCR confirmation using oLGB138 and oLGB137	This study

Name	Genotype	Construction details	Reference/Source
*Bd*CL03ΩpLGB28	*B. dorei* CL03T12C01 with pLGB28 integrated at *attBT2* site	Conjugal transfer of pLGB28 into B. dorei CL03T12C01, PCR confirmation using oLGB138 and oLGB137	This study
*Bd*CL06ΩpLGB28	*B. dorei* CL06T03C031 with pLGB28 integrated *at attBT2* site	Conjugal transfer of pLGB28 into B. dorei CL06T03C031, PCR confirmation using oLGB138 and oLGB137	This study
*Bo*CL06ΩpLGB28	*B. ovatus* CL06T03C20 with pLGB28 integrated at *attBT2* site	Conjugal transfer of pLGB28 into B. ovatus CL06T03C20, PCR confirmation using oLGB138 and oLGB137	This study
*Bo*CL09ΩpLGB28	*B. ovatus* CL09T03C03 with pLGB28 integrated *at attBT2* site	Conjugal transfer of pLGB28 into B. ovatus CL09T03C03, PCR confirmation using oLGB138 and oLGB137	This study
*Bs*CL09ΩpLGB28	*B. stercoris* CL09T03C01 with pLGB28 integrated at *attBT2* site	Conjugal transfer of pLGB28 into B. stercoris CL09T03C01, PCR confirmation using oLGB138 and oLGB155	This study
*Bt*CL06ΩpLGB28	*B. thetaiotaomicron* CL06T03C18 with pLGB28 integrated at *attBT2* site	Conjugal transfer of pLGB28 into B. thetaiotaomicron CL06T03C18, PCR confirmation using oLGB138 and oLGB137+oLGB139	This study
*Bt*CL11ΩpLGB28	*B. thetaiotaomicron* CL11T00C24 with pLGB28 integrated at *attBT2* site	Conjugal transfer of pLGB28 into B. thetaiotaomicron CL11T00C24, PCR confirmation using oLGB138 and oLGB137+oLGB139	This study
*Bu*CL12ΩpLGB28	*B. uniformis* CL12T12C23 with pLGB28 integrated at *attBT2* site	Conjugal transfer of pLGB28 into B. uniformis CL12T12C23, PCR confirmation using oLGB138 and oLGB155	This study
*Bv*CL06ΩpLGB28	*B. vulgatus* CL06T03C24 with pLGB28 integrated at *attBT2* site	Conjugal transfer of pLGB28 into B. vulgatus CL06T03C24, PCR confirmation using oLGB138 and oLGB137	This study
*Bv*CL09ΩpLGB28	*B. vulgatus* CL09T03C04 with pLGB28 integrated *at attBT2* site	Conjugal transfer of pLGB28 into B. vulgatus CL09T03C04, PCR confirmation using oLGB138 and oLGB137	This study
*Bv*CL11ΩpLGB28	*B. vulgatus* CL11T00C01 with pLGB28 integrated at *attBT2* site	Conjugal transfer of pLGB28 into B. vulgatus CL11T00C01, PCR confirmation using oLGB138 and oLGB137	This study
*Pm*CL09ΩpLGB28	*P. merdae* CL09T00C40 with pLGB28 integrated at *attBT2* site	Conjugal transfer of pLGB28 into P. merdae CL09T00C40, PCR confirmation using oLGB138 and oLGB154	This study
*Bv*CL11ΩpLGB32	*B. vulgatus* CL11T00C01 with pLGB32 integration	Conjugal transfer of pLGB32 into B. vulgatus CL11T00C01, PCR confirmation using oLGB128 and oLGB129	This study
*Bo*CL09ΩpLGB32	*B. ovatus* CL09T03C03 with pLGB32 integration	Conjugal transfer of pLGB32 into B. ovatus CL09T03C03, PCR confirmation using oLGB128 and oLGB129	This study
LGB43	*B. finegoldii* CL09T03C10, deletion of the GA1 T6SS (HMPREF1057_01517 to HMPREF1057_01551)	Conjugal transfer of pLGB18 into B. finegoldii, counterselection, PCR confirmation using oLGB33 and oLGB34 (set 1+2) or oLGB43 and oLGB164 (set 3+4)	This study

Name	Genotype	Construction details	Reference/Source
LGB82	*B. ovatus* CL09T03C03 Δ*tetQ* (AA414_04155)	Conjugal transfer of pLGB32 into B. ovatus, counterselection, PCR confirmation using oLGB128 and oLGB129	This study
LGB83	*P. merdae* CL09T00C40 with CTn341 excision	Conjugal transfer of pLGB33 into P. merdae, counterselection, PCR confirmation using oLGB128 and oLGB129 or oLGB160 and oLGB161	This study
LGB84	*B. fragilis* NCTC 9343 Δ*ubb* (BF9343_3779)	Conjugal transfer of pLGB34 into B. fragilis, counterselection, PCR confirmation using oLGB145 and oLGB146	This study
LGB85	*B. vulgatus* CL10T00C06 ΔCK234_00400-00401	Conjugal transfer of pLGB35 into B. vulgatus, counterselection, PCR confirmation using oLGB149 and oLGB150	This study
LGB86	*B. fragilis* DS_166 with pLGB37 integrated at *attBT2* site	Conjugal transfer of pLGB37 into *B. fragilis*, PCR confirmation using oLGB169 and oLGB170	This study
LGB87	*B. fragilis* US326 with pLGB37 integrated at *attBT2* site	Conjugal transfer of pLGB37 into *B. fragilis*, PCR confirmation using oLGB169 and oLGB170	This study
LGB88	*B. fragilis* S36_L11 with pLGB37 integrated at *attBT2* site	Conjugal transfer of pLGB37 into *B. fragilis*, PCR confirmation using oLGB169 and oLGB170	This study

References

Cato EP, Johnson JL.(1976) Reinstatement of species rank for *Bacteroides fragilis*, *B. ovatus*, *B. distasonis*, *B. thetaiotaomicron*, and *B. vulgatus*: Designation of neotype strains for *Bacteroides fragilis* (Veillon and Zuber) Castellani and Chalmers and *Bacteroides thetaiotaomicron* (Distaso) Castellani and Chalmers. *Int. J. Syst. Bacteriol*. 26: 230-237.

Zitomersky,N.L., Coyne,M.J. and Comstock,L.E. (2011) Longitudinal Analysis of the Prevalence, Maintenance, and IgA Response to Species of the Order Bacteroidales in the Human Gut. *Infection and Immunity*, **79**, 2012–2020.
